# Implementation of the external cephalic version in breech delivery. Dutch national implementation study of external cephalic version

**DOI:** 10.1186/1471-2393-10-20

**Published:** 2010-05-10

**Authors:** Floortje Vlemmix, Ageeth N Rosman, Margot AH Fleuren, Marlies EB Rijnders, Antje Beuckens, Monique C Haak, Bettina MC Akerboom, Joke MJ Bais, Simone MI Kuppens, Dimitri N Papatsonis, Brent C Opmeer, Joris AM van der Post, Ben Willem J Mol, Marjolein Kok

**Affiliations:** 1 Department of Gynaecology and Obstetrics, Academic Medical Centre, Amsterdam, the Netherlands; 2 Department Prevention and Health, TNO Quality of life, Leiden, the Netherlands; 3 KNOV, Leiden, the Netherlands; 4 Department of Gynaecology and Obstetrics, VU medical centre, Amsterdam, the Netherlands; 5 Department of Gynaecology and Obstetrics, Albert Schweitzer Hospital, Dordrecht, the Netherlands; 6 Department of Gynaecology and Obstetrics, Medical Centre Alkmaar, Alkmaar, the Netherlands; 7 Department of Gynaecology and Obstetrics, Catharina hospital, Eindhoven, the Netherlands; 8 Department of Gynaecology and Obstetrics, Amphia hospital, Breda, the Netherlands; 9 Department of Clinical epidemiology and biostatistics, Academic Medical Centre, Amsterdam, the Netherlands

## Abstract

**Background:**

Breech presentation occurs in 3 to 4% of all term pregnancies. External cephalic version (ECV) is proven effective to prevent vaginal breech deliveries and therefore it is recommended by clinical guidelines of the Royal Dutch Organisation for Midwives (KNOV) and the Dutch Society for Obstetrics and Gynaecology (NVOG). Implementation of ECV does not exceed 50 to 60% and probably less.

We aim to improve the implementation of ECV to decrease maternal and neonatal morbidity and mortality due to breech presentations. This will be done by defining barriers and facilitators of implementation of ECV in the Netherlands. An innovative implementation strategy will be developed based on improved patient counselling and thorough instructions of health care providers for counselling.

**Method/design:**

The ultimate purpose of this implementation study is to improve counselling of pregnant women and information of clinicians to realize a better implementation of ECV.

The first phase of the project is to detect the barriers and facilitators of ECV. The next step is to develop an implementation strategy to inform and counsel pregnant women with a breech presentation, and to inform and educate care providers. In the third phase, the effectiveness of the developed implementation strategy will be evaluated in a randomised trial. The study population is a random selection of midwives and gynaecologists from 60 to 100 hospitals and practices. Primary endpoints are number of counselled women. Secondary endpoints are process indicators, the amount of fetes in cephalic presentation at birth, complications due to ECV, the number of caesarean sections and perinatal condition of mother and child. Cost effectiveness of the implementation strategy will be measured.

**Discussion:**

This study will provide evidence for the cost effectiveness of a structural implementation of external cephalic versions to reduce the number of breech presentations at term.

**Trial Registration:**

Dutch Trial Register (NTR): 1878

## Background

Breech presentation occurs in 3 to 4% of all term pregnancies [1]. In pregnancies complicated by breech presentation, perinatal mortality, neonatal mortality or serious neonatal morbidity are increased as compared to pregnancies where the fetes is in cephalic position [2]. Breech position at term occurs in 8.000 pregnancies in the Netherlands each year. Until 2000 the mode of breech delivery was controversial. Since publication of the results of the term breech trial, the caesarean delivery rate in women with a fetes in breech presentation in the Netherlands has increased from 45% to around 80% [3]. Although an elective caesarean section is safer for the baby, it increases maternal morbidity [4]. Moreover, the uterine scar carries a risk for future pregnancies [5]. External cephalic version (ECV) reduces the rate of non-cephalic presentations at term with 40-50%, and thus the number of caesarean deliveries performed for at term breech presentation, without any increased risk to the baby [6]. The high caesarean delivery rate for breech presentation makes ECV an important obstetric intervention and it is therefore recommended by the Royal College of Obstetricians and Gynaecologists in the Clinical Green Top Guidelines.

A recent inventory among Dutch gynaecologists showed that 5% of the practices never offered ECV, whereas one practice offered ECV only to multipara. In 28% of the practices an ECV was performed by all gynaecologists, whereas in the other 72% ECV was performed by a smaller team of specialists. The palette of relative contraindications differed widely, and only 19% of the responding clinics registered their success rates. The number of patients refusing ECV was estimated to be 20 to 30%. At present, 60 to 70% of the women with a fetes in breech position undergoes ECV in the Netherlands. This rate is probably an overestimation, as they are based on self reported rates by midwives and gynaecologists, in the absence of registrations. The true number of women that undergoes ECV for term breech is probably below 50%.

With 6.000 term breech deliveries each year, of which 5.000 are delivered by caesarean section, there is clearly room for improvement. The number of caesarean sections for breech delivery can de reduced with approximately 2.000 per year. As the additional costs of a caesarean section as compared to vaginal delivery are estimated to be 1.500 Euros, the potential saving of a better implementation of ECV can reduce costs with 2 to 3 million Euros per year for direct medical costs only. As pregnant women with a previous caesarean section are at increased risk of complications, and therefore always deliver under responsibility of a gynaecologist, the potential savings are even higher.

In the Netherlands, there is professional consensus among the Dutch Society for Obstetrics and Gynaecology (NVOG) as well as the Royal Dutch Organisation for Midwives (KNOV) that ECV should be offered to all women with a fetes in breech presentation at term. The KNOV has produced a leaflet for patients in which ECV is recommended. Nevertheless, the number of women potential suitable for ECV who were not offered an attempt range from 4% to 33% [7-9]. Moreover, a substantial number of women refuse ECV, and opt immediately for a caesarean section. In Australia, Raynes-Greenow and others investigated pregnant women's preferences and knowledge of term breech management [10]. 39% would choose ECV, 39% would not choose ECV, and the remaining 22% were uncertain. Yogev and colleagues performed a similar study in Israel [11]. They reported that in 1995, more than half the women (52.7%) had heard of ECV and 53.8% were willing to consider it, whereas in 2001, 73.2% had heard of it but only 23.9% were willing to consider it. Johanson reported that out of a group of 323 pregnant women with a fetes in breech presentation, 65% opted for external cephalic version after they were informed [12]. They also demonstrated an association between the gynaecologists who provided information and the level of uptake by the women.

The number of women with a breech presentation considering ECV and their knowledge about the procedure are unknown in the Netherlands. Until publication of the new NVOG and KNOV guidelines in 2001 and 2002, ECV used to be a controversial intervention. Since the introduction of these guidelines, there is no evidence for a raise in the number of ECVs performed. However, the number of birth in breech presentation at term is stable since 2002, indicating a low implementation grade of ECV.

Implementation requires a clear and deliverable evidence based message [13]. However, there are often major discrepancies between best evidence and practice, resulting in a large variation between professionals [14-17]. Based on this national and international literature, two main barriers for ECV implementation are identified 1) lack of patients' knowledge about risk for and consequences of ECV and breech delivery 2) the attitude and knowledge of midwives and gynaecologists towards ECV.

This study will show us if an implementation strategy, tailored on main barriers combining both patient and health care provider interventions, is more cost-effective than usual care. We will also evaluate the process of implementation to ascertain which elements of the strategy can be particularly associated with successful implementation of ECV. Moreover, our proposal is a collaborative effort of midwives and gynaecologists, and is supported by both the KNOV and NVOG.

## Methods/Design

### Aims

The main objective of this study is to assess barriers and facilitators of implementation of ECV in the Netherlands. We would like to create a broad social basis and awareness for the need of cooperation in care for women with a fetes in breech position. An innovative implementation strategy will be developed based on improved patient counselling and thorough instructions of health care providers for counselling.

Research questions are: What are barriers and facilitators of implementation of ECV in the Netherlands? What are the costs and effects of an innovative implementation strategy based on improved patient counselling and information of health care providers to implement the guidelines on ECV for breech presentation? What is the feasibility of this implementation strategy?

### Methods

The proposal will contain three phases. In the first phase, we will identify facilitators and barriers of implementation of ECV and create awareness of the need for improvement. Subsequently, we will develop an implementation strategy targeted on patient counselling and information of health care providers, and evaluate the cost-effectiveness of the developed strategy.

### Identification of facilitators and barriers of implementation of ECV

A topic list will be defined by an expert panel. The topic list will be constructed on the basis of existing knowledge and theories on implementation factors, primarily based on the clinical guidelines by the NVOG and KNOV. This list will be used to guide and direct semi-structured interviews and focus groups, patients, gynaecologists and midwives will be asked for their attitude towards ECV. We will assess fear for complications, skills and attitudes. A framework approach will be used to analyse the data and to derive potential factors that might influence the implementation of ECV [18].

In the second step the exploratory findings will be used to develop a series of questionnaires. These will then be distributed among a much larger number of actors. Quantitative analyses will be conducted, accounting for the multilevel nature of the problem and the corresponding data-collection, to study the prevalence of factors that facilitate and/or inhibit the implementation of external cephalic version in the Netherlands. In this process, we will take into account regional and socio-economic differences. To identify and explore relevant factors, semi-structured interviews will be held with pregnant women with a fetes in breech presentation (N = 20), general practitioners (N = 20), midwives (N = 20), gynaecologists (N = 20), and representatives of health care insurers. Respondents will be 'purposively' selected [19]. Gynaecologists and midwives will be selected on relevant characteristics of their practices (region, hospital). Moreover, we will interview partners of pregnant women separately, but also as a couple. The interviews will be guided by the topic list that is defined on the basis of existing knowledge and theories on implementation [20]. The topic list will be used to direct the interviews, but will be informally and flexible applied in order to prevent us from imposing our preconceptions. The topic list will contain themes that include knowledge of the complications of ECV, about the risks of caesarean section in terms of short- and long term maternal and neonatal mortality and morbidity, and about available evidence. We will ask couples about their attitude towards the problem, about their expectations and previous experiences, and about their beliefs about health care in general. Midwives and gynaecologists will be interviewed on similar subjects, but they will be asked more extensively about the potential effectiveness of ECV (relative risks, numbers needed to treat, numbers needed to harm) as well as the influence of wider societal, organisational, financial, and contextual factors. We expect the following categories of factors to play a role: socio-political factors (opinion stated by opinion leaders, comments in journals, opinion of experts within the own department, attitude of the head of the department), organizational factors, and knowledge and beliefs (doctor and patient level, (the lack of) knowledge, about complications of vaginal breech delivery and caesarean section, anxiety for ECV and the demand for caesarean section (patient level only)). The semi-structured interviews will be analyzed on the basis of a framework approach [18]. The analyses will help us identify the important factors that are most likely to play a role in the implementation of ECV. We expect that we will be able to distil a list of approximately fifteen issues that are of potential importance. The exploratory findings will be used to develop a questionnaire, which is supposed to be extensive and complete. The questionnaire will be sent to all midwives practices and to the departments of obstetrics and gynaecology of all hospitals in the Netherlands. Moreover, all health insures will be targeted.

Ethical approval is not needed in accordance to the prescriptions of the medical ethical committee of the AMC, if individual patients will be approached for semi structured interviews, without provocation of emotional arousal and with anonymous processing of the data study.

### Development of an implementation plan

After analysis, the results will be discussed in a final meeting with the study group and representative participants (clients, midwives, gynaecologists). Based on the discussion of these results, an implementation plan, with different actions and interventions, will be developed. Although parts of the plan will highly depend on the obstacles of change to be identified (e.g. knowledge, attitude, behaviour, logistics), we anticipate the following ingredients: 1) an educational brochure/website with evidence, one designed for gynaecologists/midwives and one designed for patients. 2) group/unit meetings to discuss the protocol, the resistance to it, and how to overcome problems in adaptation. 3) if necessary, a training in counselling patients on ECV for midwifes and/or gynaecologists can be developed 4) reminders; regular self monitoring; regular observation by heads of units. This plan will be developed according to phase 2 to 5 of Grols 5-step implementation model. Depending on the results of the proposed project, additional funding will be sought for these phases.

### Evaluation of the developed implementation strategies design

A cluster randomised controlled trial with an economic evaluation will be performed alongside. Ethical approval will be requested before the start of this part of the study. Information on counselling of patients, required for ethical approval, will be specified in the first phase of the trial. A set of quality indicators will be extracted from the NVOG and KNOV guidelines. The indicator development will be performed according to the RAND-modified Delphi method [21]. First of all, key recommendations from the guidelines will be extracted by two or three experts, the project leaders. Subsequently, the clinical relevance of all key recommendations for patient's health benefit and efficacy will be tested in two rounds among an independent panel of 12-15 experts (KNOV and NVOG guideline writers, KNOV and NVOG members, quality of care experts and patients). The key recommendations with the highest scores will be selected and made ready for use, in measurable elements (process indicators). Moreover, measurement instruments will be developed and the participating hospitals will be informed about the study. A detailed protocol on the randomised controlled trial will be written after finishing the first phase.

This is a requirement to receive further funding of the further project. This protocol is in development; it depends mainly in the outcome of the first phase. This will be followed by a pilot, feasibility study where two different implementation strategies will be tested in four participating hospitals, one primary care centre specialised in ECV and one independent midwifery practice. Before the pilot study starts, baseline characteristics and primary and secondary outcomes will be measured with questionnaires and data gathering on amount of version attempts, outcomes after ECV and caesarean section rate. The data gathering lists and questionnaires will also be developed during the first phase.

After the pilot study a cluster randomised controlled trial will be performed in which two implementation strategies will be evaluated: a patient centred strategy and a health care provider (midwife and gynaecologist) centred strategy. In the randomised clinical trial, we will allocate centres and their regions at random to four groups: A. Patient centred strategy alone, B. Healthcare providers-centred strategy alone, C. Both the patient and healthcare provider centred strategies, D. No specific implementation intervention.

Up to 30% of eligible patients are not offered an ECV attempt and up to 40% of counselled patients do not choose for ECV, which might be the result of insufficient information. With the decision aid we intend to increase the number of well informed patients from 50 to 80%. To correct for the degree of similarity among responses within a cluster, a intra-cluster correlation coefficient of 0.10 was integrated in the power analyses. To be able to show the difference of 30% with a power of 80% and an alpha error of 5%, we need 20 clusters of a 30 patients each. The gynaecologists in the participating hospitals will give informed consent for this trial to confirm that they will treat their patients conform one of the four strategies were they will be allocated to, as long as the trial is open for inclusion.

### Outcome measures and process indicators

Primary endpoint is the number of patients that has an ECV performed. Secondary endpoints are guidelines' adherence rates, complications of ECV, the number of fetes that are in cephalic position at delivery, the number of caesarean sections and the perinatal condition of mother and child. Moreover, we will assess patients' knowledge (e.g. ECV, breech delivery, caesarean section), patients' decisional conflict and patients' satisfaction. We will also calculate costs of both implementation interventions and medical interventions. In case one or both implementation interventions are effective, their cost-effectiveness will be assessed.

### Implementation study

An effect and process evaluation will be performed. An effect evaluation of the two strategies will be carried out using the primary and secondary outcome measures and the set of process indicators derived from the NVOG and KNOV guidelines. This will be done among pregnant women with breech presentation at term and professionals involved in the care for these women. The measurements will be performed in the 20 participating hospitals before and after implementation of the different strategies by a medical record search, added with questionnaires among professionals and patients. The medical records will be searched using standardised registration forms. A process evaluation will be performed to study the feasibility of the two strategies. The extent by which clinicians, midwives and patients used these elements will be measured.

## Abbreviations

(ECV): External cephalic version; (NVOG): Dutch Society for obstetrics and gynaecology; (KNOV): The royal Dutch organisation for midwives.

## Competing interests

The study is sponsored by ZonMW; the Dutch organisation for health research and innovation. The author(s) declare that they have no competing interests.

## Authors' contributions

BWJM, MK, JAMvdP, MAHF, AB and MEBR were involved in conception and design of the study. BWMJ, MK, BCO and FV drafted the first manuscript. All authors mentioned in the manuscript are members of the 'ECV implementation study group'. They participated in the design of the study during several meetings and are local investigators in the participating centers. All authors read and approved the final manuscript.

## Authors' information

BWJM (clinical epidemiologist and gynaecologist) has been involved in many projects in the field of Obstetrics and Gynaecology. His thesis, which focused on the evaluation of diagnostic and prognostic tests in sub fertility, was awarded with the Jan Swammerdam prize. In 2002, he has initiated the Dutch OFO-project, a study that aims to evaluate the effectiveness and cost effectiveness of the basic fertility work-up. The key paper of this project has been published in The Lancet (Lancet 2006;368:216-21). A study proposal entitled: Use of probabilistic decision rules in Obstetrics and Gynaecology was granted in the VIDI program of ZonMw, the Netherlands. He has (co-)authored over 150 peer reviewed publications, and he has supervised nine doctorates. He was instrumental in founding a Dutch consortium of obstetricians that cooperates in performing studies in obstetrics http://www.studies-obsgyn.nl/index.asp. He is chair of the Dutch guideline committee of the NVOG. He is involved in implementation studies in obstetrics.

PMO practiced ten years in several independent midwifery practices. In 2001 she graduated in Health Science at the University of Maastricht. From 1995 until 2003 she worked as midwife researcher in several projects, at TNO-PG and later at WOK (UMCN), e.g. a study to assess the adherence and barriers for implementation of the KNOV guideline Anemia for primary care midwifes. Since 2003 she is staff member of the Royal Dutch Organisation for Midwifes (KNOV), and there she is responsible for the guideline development. In 2006 she was co-auteur of the KNOV guideline 'External Cephalic Version'. Offerhaus P, Fleuren M, Wensing M. Guidelines on anaemia: effect on primary care midwives in the Netherlands. Midwifery 2005;(21): 204-211

MR works as a research-midwife at TNO quality of Life. She has extensive experience in conducting research projects in independent midwifery practices: for example the Serinam study, a RCT in 55 midwifery practices, the ECV prevalence study in 50 midwifery practices (both still ongoing) and a retrospective study on satisfaction and mode of delivery in 8 midwifery practices. Furthermore, she conducted 2 studies on ECV: a retrospective study of all ECV between 1996 and 2000 in the Slotervaart Hospital and a prospective study on ECV in the same hospital including expectations and experiences if women with ECV (publication in press). Other research projects she participated in were a cost effectiveness study on screenings strategies for EOGBS and a study on information and informed consent of women with neonatal screening. Finally she was co author of the first midwifery guideline "Anaemia in first line obstetric practice".

JAMvdP is clinical director of obstetrics in the AMC and his main research focus is high risk pregnancy. His thesis dealt with pathofysiology of pre-eclampsia. He is currently involved in management of the Perinatal Research Unit, a collaborative initiative from the departments of obstetrics and neonatology for the monitoring of multicenter clinical studies, and a consortium of perinatal centres in the Netherlands. Work in progress concerns early diagnosis of pre-eclampsia, external version for breech presentation (RCT) and treatment of recurrent abortion (RCT), the value of the intrauterine pressure catheter IUPC trial (RCT).

BCO (psychologist, methodologist) has participated in the coordination of European collaborative studies (European Communities 4th Medical and Health Research Program COMAC; BIOMED1) concerning health services research and is since 1999 working in the department of Clinical Epidemiology and Biostatistics in the Academic Medical Center. He has been involved in several evaluation studies (RCTs) in the fields of Dermatology, Surgery and Infectious diseases respectively, especially focusing on the economic and methodological perspective. He has conducted a study on methods for determining patients' treatment preferences and trade-offs in health care technology assessment research (ZONMW 2001-2003). He is currently involved in several studies in the field of obstetrics, gynaecology and fertility research.

## Pre-publication history

The pre-publication history for this paper can be accessed here:

http://www.biomedcentral.com/1471-2393/10/20/prepub
